# PRO: The COVID-19 pandemic will result in increased antimicrobial resistance rates

**DOI:** 10.1093/jacamr/dlaa049

**Published:** 2020-07-17

**Authors:** Cornelius J Clancy, Deanna J Buehrle, M Hong Nguyen

**Affiliations:** University of Pittsburgh, Department of Medicine, Pittsburgh, PA, USA; VA Pittsburgh Healthcare System, Pittsburgh, PA, USA; VA Pittsburgh Healthcare System, Pittsburgh, PA, USA; University of Pittsburgh, Department of Medicine, Pittsburgh, PA, USA

## Abstract

We argue that the COVID-19 pandemic will result in increased antimicrobial resistance (AMR). Broad-spectrum antibiotic use is common among hospitalized COVID-19 patients and in excess of reported secondary infection rates, suggesting unnecessary prescribing. Selection pressure is likely to be particularly intense in COVID-19 epicentres and within non-epicentre hospital units dedicated to COVID-19 care. Risk factors that increase the likelihood of hospitalization or poor outcomes among COVID-19 patients, such as advanced age, nursing home residence, debilitation, diabetes and cardiopulmonary or other underlying systemic diseases, also predispose to AMR infections. Worry for AMR emergence is heightened since first-wave COVID-19 epicentres were also AMR epicentres. Disruptive direct and indirect effects of COVID-19 globally on economic systems, governance and public health expenditure and infrastructure may fuel AMR spread. We anticipate that the impact of COVID-19 on AMR will vary between epicentres and non-epicentres, by geographic region, hospital to hospital within regions and within specific hospital units.

In recent years, repeated warnings have been sounded about public health threats posed by pandemic viral and antimicrobial resistant (AMR) infections.^1^^,^^2^ The world is now experiencing a pandemic of coronavirus disease 2019 (COVID-19) due to the novel severe acute respiratory syndrome coronavirus-2 (SARS-CoV-2), which arose in Wuhan, China in late 2019.^3^ AMR infections are conservatively estimated to cause 700 000 deaths annually worldwide, a number that is projected to increase to 10 million per year by 2050.^4^ There has been much speculation, but as yet no conclusive data, about how or whether the threats of COVID-19 and AMR may come together. In this paper, we argue that the COVID-19 pandemic will result in increased AMR.

## COVID-19 and AMR: what do we know?

Detailed information on secondary bacterial or fungal infections among patients hospitalized with COVID-19 is sparse.^3^ Most reports of COVID-19 that have mentioned secondary infections have done so in passing, without providing case definitions, diagnostic criteria or methods for distinguishing colonization from disease. Secondary infections have been reported in ∼10% of COVID-19 patients included in published series from hospitals in China, Europe and the USA, with incidence as high as 35% among ICU residents.^3^^,^^5^ The most common type of infection was pneumonia (especially ventilator-associated pneumonia), followed by bloodstream and urinary tract infections. Certain centres, mostly in Europe, have reported incidence of invasive aspergillosis as high as 20%–30% among critically ill COVID-19 patients.^6^ In autopsy studies of SARS-CoV-2-infected persons, histopathologic findings consistent with bacterial bronchopneumonia have been reported in about 30% of cases but, with rare exception, culture data were lacking.^6–8^

**Figure 1.**
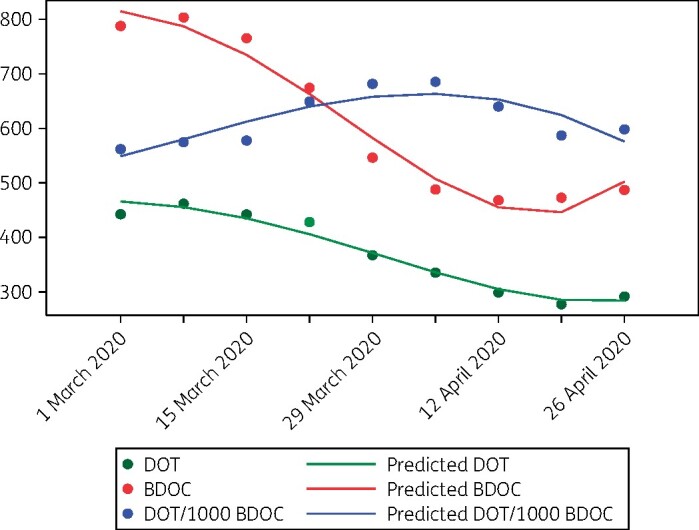
Weekly in-hospital antibiotic use and BDOC in a healthcare system outside of a COVID-19 epicentre, 1 March to 2 May 2020. Data are presented as 3 week rolling averages of numbers of in-hospital antibiotic DOT, BDOC and DOT/1000 BDOC (*y*-axis) each week (*x*-axis; dates represent the first day of a given week). In-hospital antibiotic DOT and BDOC per week decreased significantly from 1 March to 2 May 2020. Note the increase in DOT/1000 BDOC. The week-to-week difference in DOT/1000 BDOC over the time period was not statistically significant. However, since antibiotic use is likely concentrated in patients with COVID-19, the data suggest that selection pressure may be markedly increased within hospital units devoted to COVID-19 care, even at centres outside a disease epicentre.

Antibiotics were administered to most hospitalized COVID-19 patients in reports to date, including 75%–100% of those in ICUs.^3^ Most commonly used agents have been fluoroquinolones, cephalosporins, carbapenems, azithromycin, vancomycin and linezolid. The impact of COVID-19 on overall antibiotic use within hospitals, healthcare systems and regions is unclear, as cancellation of non-essential medical services, surgeries and outpatient clinic visits has led to sharp decreases in the numbers of non-COVID-19 patients seeking care. Sparse data since the onset of the pandemic suggest significant reductions in antibiotic prescriptions. A news report cited unpublished data from IQVIA (Durham, NC, USA) that described a 45% drop in antibiotic purchases in the USA between the start of January and mid-April 2020.^7^ Estimated total prescription fills for amoxicillin and azithromycin, the most commonly prescribed antibiotics in the USA, were each down nationally by over 60% in the week of 19–25 April 2020 compared with the same week in 2019.^8^ In our healthcare system in western Pennsylvania, removed from a disease epicentre, in-hospital antibiotic days of therapy (DOT) and outpatient antibiotic prescriptions decreased by an average of 5.1% and 6.1% per week, respectively, between 1 March and 2 May 2020 (Figure 1) (D. J. Buehrle, B. K. Decker, M. M. Wagener, A. A. Adalja, N. Singh, M. C. McEllistrem, M. H. Nguyen and C. J. Clancy, unpublished data). In-hospital antibiotic use lagged behind reductions in bed days of care (BDOC) by a week.

Limited microbiological data published during the COVID-19 pandemic suggest that causative organisms and AMR patterns for secondary infections were consistent with institutional ecology. In reports from China, organisms included pandrug-resistant (PDR) *Acinetobacter baumannii*, KPC-producing *Klebsiella pneumoniae*, ESBL-producing *K. pneumoniae*, ESBL-producing *Pseudomonas aeruginosa*, MRSA, *Aspergillus fumigatus*, *Aspergillus flavus*, *Candida albicans* and *Candida glabrata*.^3^ Anecdotal evidence for the potential of COVID-19 to accelerate AMR appeared in a *British Medical Journal* news feature, in which a stewardship director described ‘a slow but steady increase in multi-drug resistance (MDR)’ at a large New York City health system.^9^ In contrast, an online viewpoint from Geneva, the canton in Switzerland with the highest incidence of COVID-19, reported that weekly screening of ICU patients showed decreased asymptomatic colonization and disease by MDR organisms.^10^ Data were not presented in either of these pieces.

## Why might AMR arise?

Clearly, no conclusions about relationships between COVID-19 and AMR can be drawn from published reports. There are several reasons, however, that we expect AMR to emerge during the pandemic.

Broad-spectrum antibiotic use has been widespread among hospitalized COVID-19 patients, and in excess of reported secondary infection rates. Therefore, much of the use to date has been unnecessary. Antibiotic therapy will likely remain common owing to difficulties in conclusively excluding secondary pneumonia or nosocomial superinfections in many cases. A large percentage of patients admitted to hospitals with COVID-19 are from congregated living facilities such as nursing homes, prisons and psychiatric hospitals.^11^ Patients from nursing homes, in particular, are more likely to have altered microbiomes and to be colonized with ESBL- and carbapenemase-producing organisms, MRSA, VRE and *Candida*. This population may drive high rates of broad-spectrum antibiotic use, especially since empiric therapy upon hospital admission will likely be directed against healthcare-associated pathogens.

COVID-19 strained hospital capacity to breaking point in hard-hit epicentres globally. As such, overall antibiotic use in these locations and at facilities in other locations that cared for large numbers of COVID-19 patients was likely up sharply, even if non-emergency hospital services were suspended or regional antibiotic trends were down. For many hospitals outside of epicentres, antibiotic use within ICUs or wings that converted into COVID-19 units was likely increased, particularly if normalized to BDOC (Figure 1). Therefore, it is reasonable to assume that there will be significant AMR selection pressure in COVID-19 units, regardless of patterns elsewhere in a facility. Hospitals and regions with high AMR prevalence that have moderate-to-high numbers of COVID-19 patients should be prepared for potential upswings in nosocomial infections by these pathogens. Risk factors and comorbidities that increase the likelihood of hospitalization or poor outcomes among COVID-19 patients, such as advanced age, debilitation, diabetes and cardiopulmonary or other underlying systemic diseases, also predispose to development of AMR infections.

Worry for emergence of AMR is heightened by the fact that first-wave COVID-19 epicentres were also AMR epicentres. As of 31 May 2020, countries with the largest numbers of diagnosed COVID-19 cases were the USA, Brazil, Russia, the UK, Spain and Italy,^12^ each of which face significant challenges with MDR Gram-negative and other AMR bacteria. New York and Lombardy, regions within the USA and Italy, respectively, that were hit disproportionately hard by COVID-19, are both AMR hotspots,^13^ as are China (where the pandemic began) and India and Mexico (large nations with among the most rapid growth in newly diagnosed COVID-19 cases at the time of writing). Local selection pressure for AMR during COVID-19 will be overlaid on larger, ongoing and highly dynamic regional trends, such as the remarkable growth of PDR *A. baumannii* and MBL-producing Enterobacteriaceae in Asia and ESBL-producing Enterobacteriaceae in the USA.^14^^,^^15^

While antibiotic exposure is undeniably a major risk factor for AMR,^16^ it is simplistic to conclude that the reductions in overall antibiotic prescriptions reported thus far will lessen the likelihood of AMR during the COVID-19 pandemic. Reduced antibiotic use has correlated with suspensions of healthcare services during a time of stay-at-home and other extreme protective measures. As restrictions are lifted and healthcare activities resume, it is likely that antibiotic prescribing will rebound. It is also notable that peaks of COVID-19 in Europe and North America occurred at the end of the 2019–20 influenza season. It is unclear what pressures will be placed on healthcare and antibiotic utilization if COVID-19 resurges during the upcoming influenza season. If antibiotics are prescribed liberally in outpatient settings, particularly as SARS-CoV-2 and influenza cocirculate, AMR may also emerge in communities. It is important to understand that any changes in human antibiotic use are small in the face of global antibiotic use, over 80% of which occurs in animal husbandry and agriculture.^17^ These sectors have generally been designated as essential and not restricted by COVID-19. Non-human antibiotic use is clearly linked to AMR in humans.^17^ As one example, *Escherichia coli* with colistin resistance due to plasmid-borne *mcr1* arose in pigs in China, which led to worldwide human dissemination in 2015 and horizontal gene transfer to other Enterobacteriaceae.^15^^,^^18^ Risks of animal-to-human transmission of novel AMR will remain and COVID-19 may increase the pool of potentially vulnerable hosts.

The volume of human antibiotic consumption has an inconsistent impact on AMR rates.^16^^,^^19^ Models using human population-based data do not reliably show causal relationships between antibiotic exposure and AMR, even if such relationships exist at the level of individual patients.^20^ Therefore, active surveillance of SARS-CoV-2-infected individuals for newly emergent AMR pathogens will be crucial. Once AMR arises (as, for example, might happen in COVID-19 patients in hospital units facing increased selection pressure, or in animals due to husbandry practices), socioeconomic and anthropological factors that drive spread are often more important determinants of prevalence than overall antibiotic consumption.^16^ In this regard, disruptive direct or indirect effects of COVID-19 on economic systems, governance, sanitation and water safety, and public health expenditures, infrastructure and regulation may fuel increasing AMR.

## What can we expect?

We anticipate that the impact of COVID-19 on AMR will be uneven, varying between epicentres and non-epicentres, by geographic region, hospital-to-hospital within regions and within specific hospital units. Initially, local AMR patterns are likely to be exacerbated. As travel resumes, the risk of dissemination of novel resistance determinants will increase. AMR screening programmes should be implemented for asymptomatic hosts and livestock following antibiotic exposure. Research protocols can evaluate the impact of antibiotic use on microbiota and resistomes, as part of more sensitive surveillance.

## Transparency declarations

None to declare.
